# Energy security performance: A dataset on the member countries of the Commonwealth of Independent States, 2000–2014

**DOI:** 10.1016/j.dib.2019.104450

**Published:** 2019-08-29

**Authors:** Svetlana Vladislavl'evna Lobova, Julia Vyacheslavovna Ragulina, Aleksei Valentinovich Bogoviz, Alexander Nikolaevich Alekseev

**Affiliations:** a Altai State University, Barnaul, Russia; b Ural State University of Economics, Ekaterinburg, Russia; c Peoples' Friendship University of Russia (RUDN University), Moscow, Russia; d National Research University Higher School of Economics, Moscow, Russia; e Financial University Under the Government of the Russian Federation, Moscow, Russia

**Keywords:** Energy security, Index, Performance, Commonwealth of Independent States

## Abstract

This paper presents a rich collection of data used to construct an energy security performance index of the eight countries of the Commonwealth of Independent States (CIS). Namely, the index covers Azerbaijan, Armenia, Belarus, Kazakhstan, Kyrgyz Republic, Russia, Tajikistan, Ukraine, Uzbekistan. The data include results of Z-score normalization of 12 distinct indicators, constituting a total of 4 dimensions of energy security performance. These dimensions are the following: energy availability (oil import dependence, coal import dependence, natural gas import dependence), energy affordability (access to electricity, pump price for gasoline, pump price for diesel fuel), energy and economic efficiency (renewable energy consumption, GDP per unit of energy use, electric power consumption), and environmental stewardship (CO_2_, N_2_O, and SO_2_ emissions). Z-scores are calculated for 2000 and 2014, which allows to evaluate energy security performance of the CIS countries in each dimension and in total over the period of 14 years.

**Table:** Specifications Table

Subject area	*Energy Economics and Policy*
More specific subject area	*Energy Security*
Type of data	*Table, figure, MS Excel file*
How data was acquired	*A dataset was constructed in order to evaluate energy security performance of the member states of the Commonwealth of Independent States (CIS) in 2000-2014. We collected 12 indicators and used them to operationalize a total of 4 dimensions of energy security: energy availability, energy affordability, energy and economic efficiency, and environmental stewardship. Indicators were obtained from the databases of international organizations (World Bank, International Energy Agency) and national statistical services of the CIS countries. Then we applied a z-score normalization on all indicators, treating them as diverse units of measurement, in order to evaluate their relative magnitudes of change. In other words, each data point of every indicator was turned into a z-score, resulting in a dimensionless quantity that indicates how many standard deviations a particular country is below or above the CIS level. Z-scores were calculated separately for 2000 and 2014 and then summed up for each year, giving equal weight for each dimension of the energy security performance index. Then, we multiplied all the variables by -1 in order to have a higher value indicating better energy security performance. Z-scores for year 2000 were subtracted from year 2014 in order to evaluate how energy security performance had changed over the period of 14 years.*
Data format	*Raw and analyzed*
Experimental factors	*Our quantitative energy security index is based on 12 numerical indicators collected from open sources, which serve as reliable proxies of energy security.*
Experimental features	*The z-scored standardization methodology was applied to calculate z-scores for each indicator and then evaluate energy security performance.*
Data source location	*National statistical services of the 8 CIS countries: Azerbaijan, Armenia, Belarus, Kazakhstan, Kyrgyz Republic, Russia, Tajikistan, Ukraine, Uzbekistan. Official databases of the World Bank and International Energy Agency.*
Data accessibility	*All data are with this article.*
Related research article	Bogoviz, A.V., Lobova, S. V., Ragulina, Yu. V., Alekseev, A. N. (2017). A comprehensive analysis of energy security in the member states of the Eurasian Economic Union, 2000–2014. *International Journal of Energy Economics and Policy,****7***(5), 93–101. URL: http://econjournals.com/index.php/ijeep/article/view/5447[1].

**Table:** 

**Value of the data** • Provides a comprehensive view on the development of energy security in the Commonwealth of Independent States (CIS) by constructing an energy security performance index. • The quantitative index consists of 4 dimensions with a total of 12 numerical indicators, which allows to analyze the changes in each dimension of energy security over the period of 14 years. • The indicators are analyzed with the method of Z-score normalization, which allows to account for “special cause variation” and evaluate how well a country performs in energy security relative to performance of other CIS countries. • The data are used for analyzing energy security performance made by the CIS member countries in 2000–2014. More than that, the dataset could be potentially easily expanded by collecting data for additional years and including them into the index. This would allow to precisely analyze energy security performance of particular CIS countries over the necessary periods of time. • The MS Excel file with all the calculations made is attached to the paper. This provides a potential opportunity for the researchers to use the existing structure of our dataset for creating an energy security performance index for any other country or a group of countries.

## 1 Data

The paper presents the data used to build an energy security performance index in order to evaluate the member countries of the Commonwealth of Independent States (CIS) from 2000 to 2014. In particular, the index covers the following countries: Azerbaijan, Armenia, Belarus, Kazakhstan, Kyrgyz Republic, Russia, Tajikistan, Ukraine, Uzbekistan.

The data cover a total of two years (2000 and 2014) and include results of the Z-score normalization made for each dimension of energy security, including energy availability, its efficiency, affordability, and environmental stewardship. Each dimension is operationalized with a total of three indicators. The next section (Experimental Design, Materials, and Methods) provides a detailed review of each index dimension and its values, as well as it describes the method for calculating z-scores.

In short, each z-score is a dimensionless unit of measurement, which represents a corresponding value of the energy security indicator and allows to evaluate its relative magnitude of change. Since our index consists of diverse units of measurement, the methodology of z-score normalization is absolutely necessary to turn them into dimensionless quantities, indicating how many standard deviations a particular country is below or above the CIS level in each dimension of energy security and in total.

**Fig. 1:**
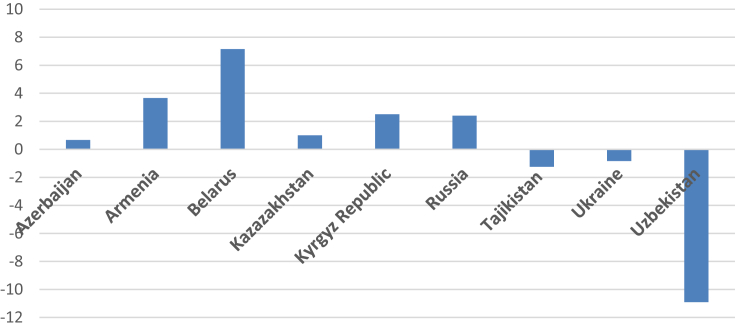
Energy security performance by the CIS member countries. 2000–2014.

**Fig. 2:**
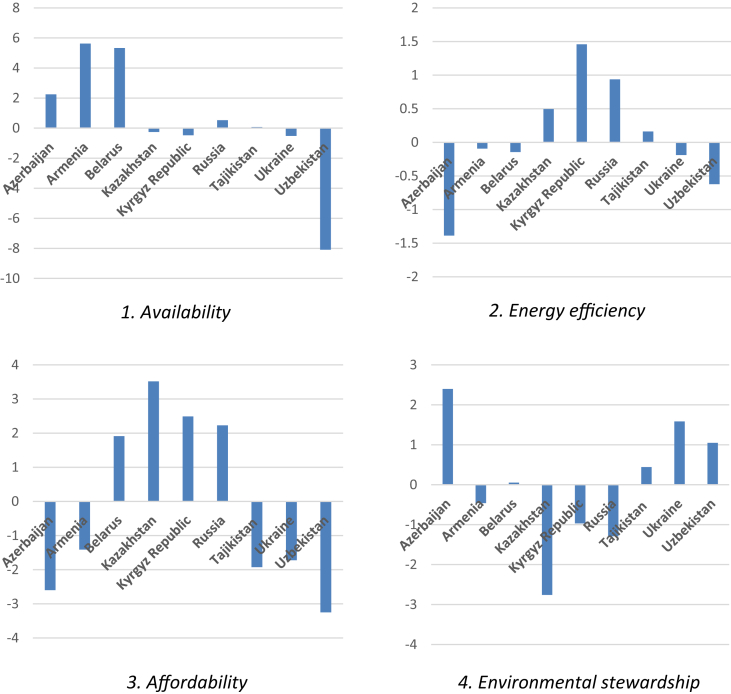
Energy security performance of the CIS countries in each dimension, 2000–2014.

Z-scores for every indicator are presented in Table 1 (2000) and 2 (2014). The energy security performance index for 2000 and 2014 is provided in Table 3, Table 4. Shifts in the values of the energy security performance index between 2000 and 2014 are presented in Table 5 (for each dimension) and Table 6 (in total). Positive values indicate better energy security performance. Graphically, the overall energy security performance is presented in Fig. 1, and a detailed performance in each dimension is available in Fig. 2 (see Table 2).

**Table 1:** Energy security indicators, Z-scores, 2000.

Country	Availability			Affordability		
	Oil import dependence, %	Coal import dependence, %	Natural gas import dependence, %	Access to electricity, % of population	Pump price for gasoline, US$ PPP/L	Pump price for diesel fuel, US$ PPP/L
Azerbaijan	1.396419314	0.092022957	−0.154121725	2.329014548	0.430190367	0.583908716
Armenia	−0.614383065	0.092022957	−6.174783064	0.171701901	0.343139531	0.508444276
Belarus	−1.472423942	−7.049990674	−0.071535196	−0.736640266	−0.940588676	−0.060405805
Kazakhstan	1.49316311	2.054837172	0.503995418	0.004794028	−0.044396723	−0.239012062
Kyrgyz Republic	−0.458590703	−1.831307407	0.062586118	−0.776380236	−0.669255081	−0.325972353
Russia	0.763845044	0.18553205	0.616571284	−0.623097495	1.05073573	0.547619559
Tajikistan	−0.470749513	−0.213961419	0.279442517	0.662206672	−1.286036187	−2.230502039
Ukraine	−0.486784955	0.06542227	0.266444559	−0.371032544	−0.183295536	−0.172823256
Uzbekistan	−0.15049529	0.112169453	6.689464637	−0.660566609	2.056138146	1.388742965
Country	Energy and economic efficiency			Environmental stewardship		
	Renewable energy consumption, % of total	GDP per unit of energy use, US$ 2011 PPP per kg oil equiv.	Electric power consumption, kWh per capita	CO_2_/GDP PPP, kg CO_2_/2010 US$	N_2_O emissions, thousand metric tons of CO_2_ equivalent	SO_2_ emissions, tones per capita

Azerbaijan	0.530612116	0.037315314	0.451619767	−0.0125	−0.646480572	0.521650593
Armenia	0.291179139	−1.108115553	1.086720537	0.813517685	0.744379536	0.57025158
Belarus	0.393612117	0.132245471	−0.365501488	0.198418948	0.376044607	0.395288027
Kazakhstan	0.509994016	−0.834967457	−0.513578909	0.059525684	0.262444896	−0.722534673
Kyrgyz Republic	−1.024011457	−1.041843556	0.746053733	0.694466316	0.711248272	0.550811186
Russia	0.463403683	−0.096124248	−2.248042582	−0.019841895	−2.351925071	−0.042120855
Tajikistan	−2.303648745	−0.188367703	0.348068842	0.992094738	0.722687074	0.560531383
Ukraine	0.568701591	1.303647796	−0.179246775	−0.396837895	−0.256678398	−2.404128822
Uzbekistan	0.570157539	1.796209936	0.673906875	−2.38102737	0.438279656	0.57025158

**Table 2:** Energy security indicators, Z-scores, 2014.

Country	Availability			Affordability		
	Oil import dependence, %	Coal import dependence, %	Natural gas import dependence, %	Access to electricity, % of population	Pump price for gasoline, US$ PPP/L	Pump price for diesel fuel, US$ PPP/L
Azerbaijan	2.08589224	0.25451084	1.24315527	0.33140543	−0.2870754	0.70125356
Armenia	−0.6193157	0.25451084	−0.7083191	0.33553569	−0.3430962	−0.3784261
Belarus	−0.7329745	−0.7685543	−1.7612745	0.33253187	0.17193884	−0.3297377
Kazakhstan	1.17906405	1.51601911	1.10587689	0.33498374	1.48874426	1.41262304
Kyrgyz Republuc	−0.589872	−1.1963298	−0.9153377	0.33240045	0.0893338	0.29576699
Russia	0.27972425	1.09576349	0.71483917	0.33290735	1.64225318	1.22865383
Tajikistan	−0.6986261	0.2241159	0.14078049	−2.6666643	−1.0918468	−1.0202653
Ukraine	−0.539337	0.13067629	−0.2583496	0.33411638	−1.1970474	−1.5869936
Uzbekistan	−0.3645552	−1.5107124	0.43862911	0.33278344	−0.4732042	−0.3228748
Country	Energy and economic efficiency			Environmental stewardship		
	Renewable energy consumption, % of total	GDP per unit of energy use, US$ 2011 PPP per kg oil equiv.	Electric power consumption, kWh per capita	CO_2_/GDP PPP, kg CO_2_/2010 US$	N_2_O emissions, thousand metric tons of CO_2_ equivalent	SO_2_ emissions, tones per capita
Azerbaijan	0.05841256	−0.9518616	0.52619961	1.17997925	0.63431322	0.44556694
Armenia	0.22994537	−0.7089474	0.6547871	1.0182562	0.71570128	−0.0648097
Belarus	0.30221685	−0.0099421	−0.2768045	0.42527171	0.08017284	0.51847789
Kazakhstan	0.65516139	0.32170568	−1.3202028	−0.8685126	−0.1130469	−2.1792274
Kyrgyz Republic	−1.0256972	0.49697005	0.6681355	−0.4372512	0.68884946	0.73721075
Russia	0.51499047	0.40625945	−1.865157	−0.1677128	−2.4500755	−1.0855631
Tajikistan	−2.2893552	−0.611259	0.91891126	1.23388693	0.67498611	0.8101217
Ukraine	0.50982349	1.12825017	−0.1347485	−1.3536818	−0.3462788	0.22683408
Uzbekistan	0.54976823	1.03959185	0.82887937	−1.0302357	0.11537832	0.59138884

**Table 3:** Energy security performance index, Z-scores, 2000.

Country	Availability	Efficiency	Affordability	Stewardship	Total
Azerbaijan	1.33432055	1.0195472	3.34311363	−0.13733	5.5596514
Armenia	−6.6971432	0.26978412	1.02328571	2.1281488	−3.2759245
Belarus	−8.5939498	0.1603561	−1.7376347	0.96975158	−9.2014769
Kazakhstan	4.0519957	−0.8385524	−0.2786148	−0.4005641	2.5342645
Kyrgyz Republic	−2.227312	−1.3198013	−1.7716077	1.95652577	−3.3621952
Russia	1.56594838	−1.8807631	0.97525779	−2.4138878	−1.7534448
Tajikistan	−0.4052684	−2.1439476	−2.8543316	2.2753132	−3.1282344
Ukraine	−0.1549181	1.69310261	−0.7271513	−3.0576451	−2.246612
Uzbekistan	6.6511388	3.04027435	2.7843145	−1.3724961	11.1032315

**Table 4:** Energy security performance index, Z-scores, 2014.

Country	Availability	Efficiency	Affordability	Stewardship	Total
Azerbaijan	3.58355836	−0.3672495	0.74558359	2.2598594	6.22175189
Armenia	−1.0731239	0.1757851	−0.3859866	1.66914775	0.38582231
Belarus	−3.2628033	0.01547018	0.174733	1.02392244	−2.0486777
Kazakhstan	3.80096005	−0.3433357	3.23635104	−3.1607869	3.53318846
Kyrgyz Republic	−2.7015395	0.13940834	0.71750124	0.98880901	−0.855821
Russia	2.09032691	−0.9439071	3.20381436	−3.7033514	0.6468828
Tajikistan	−0.3337297	−1.981703	−4.7787765	2.71899474	−4.3752145
Ukraine	−0.6670103	1.50332512	−2.4499246	−1.4731265	−3.0867363
Uzbekistan	−1.4366385	2.41823944	−0.4632955	−0.3234685	0.19483684

**Table 5:** Shifts in the energy security performance index, Z-scores, 2014–2000 (dimensions).

Country	Availability	Efficiency	Affordability	Stewardship
Azerbaijan	2.24923781	−1.3867967	−2.59753	2.39718938
Armenia	5.62401924	−0.093999	−1.4092723	−0.4590011
Belarus	5.33114653	−0.1448859	1.91236775	0.05417086
Kazakhstan	−0.2510356	0.49521664	3.5149658	−2.7602228
Kyrgyz Republic	−0.4742276	1.45920962	2.48910891	−0.9677168
Russia	0.52437854	0.93685609	2.22855656	−1.2894636
Tajikistan	0.0715387	0.16224461	−1.9244449	0.44368154
Ukraine	−0.5120922	−0.1897775	−1.7227733	1.58451864
Uzbekistan	−8.0877773	−0.6220349	−3.24761	1.04902761

**Table 6:** Shifts in the energy security performance index, Z-scores, 2014–2000 (total).

Country	2000	2014	Total
Azerbaijan	5.5596514	6.22175189	0.6621005
Armenia	−3.2759245	0.38582231	3.66174685
Belarus	−9.2014769	−2.0486777	7.15279922
Kazakhstan	2.5342645	3.53318846	0.99892396
Kyrgyz Republic	−3.3621952	−0.855821	2.50637421
Russia	−1.7534448	0.6468828	2.4003276
Tajikistan	−3.1282344	−4.3752145	−1.2469801
Ukraine	−2.246612	−3.0867363	−0.8401243
Uzbekistan	11.1032315	0.19483684	−10.908395

The data obtained allow to evaluate energy security performance made by each CIS member country by looking into changes in relevant z-scores. For instance, the data clearly show that Azerbaijan, Armenia, Belarus, Kazakhstan, Kyrgyz Republic, and Russia have improved their energy security performance since 2000, with the best results achieved by Belarus (+7.15) and Armenia (+3.66). In turn, other three CIS countries (Tajikistan, Ukraine, and Uzbekistan) have lowered their energy security, with the worst performance demonstrated by Uzbekistan (−10.9).

## 2 Experimental Design, materials, and methods

**Fig. 3:**
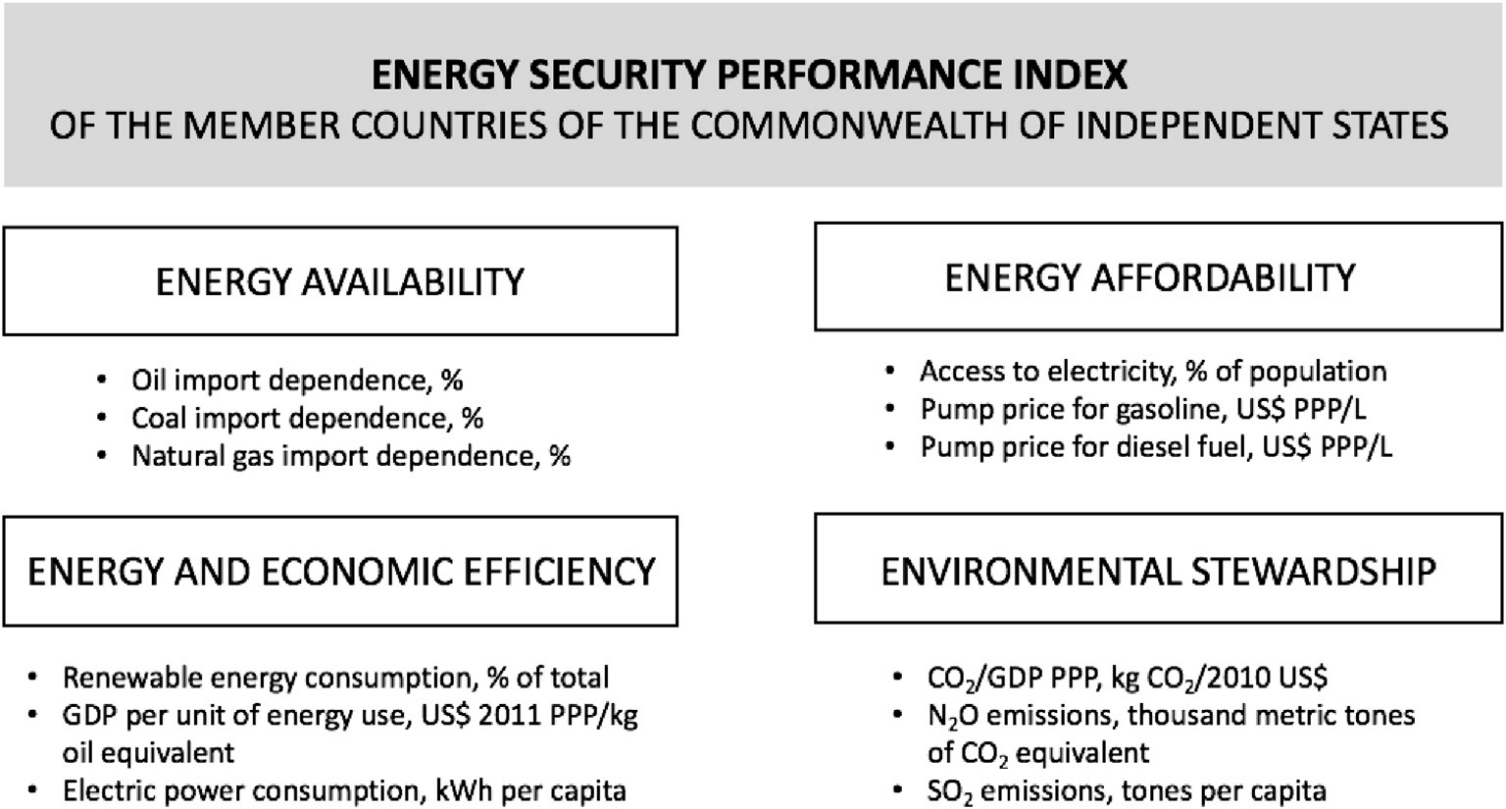
Dimensions and indicators of the energy security performance index.

The energy security performance index of the CIS member countries is based on the concept of energy security developed by Sovacool and Brown [2], [3], [4]. In particular, energy security is seen as a combination of four dimensions: energy availability, energy affordability, economic and energy efficiency, and environmental stewardship. To construct a quantitative index, we operationalize each dimension with a total of three numerical indicators, which serve as reliable proxies and are available for all CIS countries in the given time period. All dimensions and relevant indicators are graphically presented in Fig. 3.

To reflect “energy availability,” we calculate dependency on three major fossil fuels (oil, coal, natural gas) for each CIS country. These fossil fuels constitute the largest part of energy consumption in this group of counties [5]. Dependency is calculated as the ratio of net imports to final products supplied [6]. Data on fossil fuel imports and consumption are obtained from the Statistical Database of the International Energy Agency [7].

We use “access to electricity,” “pump price for gasoline,” and “pump price for diesel fuel” as reliable indicators to measure the “energy affordability” dimension of the energy security performance made by the CIS countries. All these indicators were obtained from the World Bank [5]. Gasoline and diesel fuel process are adjusted to PPP by us, with the purpose of obtaining a set of comparable values.

The “energy and economic efficiency” dimension is operationalized using the following indicators: “renewable energy consumption,” “GDP per unit of energy use,” and “electric power consumption.” These indicators are obtained from the World Bank Database [5].

Lastly, to reflect the “environmental stewardship” dimension, we focus on greenhouse gas emissions: CO_2_, N_2_O, and SO_2_. The national statistical services provided information on SO_2_ emissions [8], [9], [10], [11], [12], [13], [14], [15], [16]. These data were available online for Russia, Ukraine, Armenia, Belarus, Kazakhstan, and Kyrgyz Republic only. With respect to Azerbaijan, Tajikistan, and Uzbekistan, we got the necessary indicators by processing official queries from the official sources of government statistics. Other indicators (CO_2_ and N_2_O emissions) were obtained from the World Bank [5].

In order to analyze the diverse units of measurement presented in our index, we apply the method of Z-score normalization. This methods traces a relative magnitude of changes in each indicator and allows to account for “special cause variation” in the data, i.e. when certain steps made by a country lead to distinct changes in energy security performance. The application of this method results in creating a dimensionless quantity for each indicator and allows to analyze how many deviations a particular country is above or below the level of other 8 CIS countries.

(1) $$\text{Z}-{\text{score}}_{a,b}=\frac{\text{absolute}{\text{value}}_{a,b}-{\text{mean}}_{a,b}}{{\text{standard deviation}}_{a,b}}$$

To calculate z-scores for all 12 indicators, we subtracted the mean value for each data point and divided by the indicator's standard deviation, following the formula (1). All the calculations are made in the MS Excel file, which is attached to this paper as Appendix A. Then we sum up z-scores in every dimension for each year and multiply them by −1. This is necessary in order to have higher indicators indicating better energy security performance. To calculate changes in energy security performance made over 14 years, we subtract z-score values for year 2000 from year 2014.
